# Supplementary material for: Hypocalcemia and Vitamin D Deficiency amongst Migraine Patients: A Nationwide Retrospective Study
Source: Medicina (Kaunas). 2019 Jul 25;55(8):407. doi: 10.3390/medicina55080407 (PMC6723741; doi:10.3390/medicina55080407)
Supplement: Supplementary file 1 [file medicina-55-00407-s001.pdf]

SUPPLEMENTAL MATERIAL

Hypocalcemia and Vitamin D Deficiency amongst Migraine Patients: A Nationwide Retrospective Study

Table S1. ICD-9-CM codes used in this analysis.

**Condition**	**ICD-9 CM Codes**
Hypercholesterolemia/lipidemia	272.0, 272.1, 272.2
Hypertension	401-405
Diabetes Mellites	249.00, 250
Chronic use of NSAIDs	V58.64
Alcohol abuse/dependent	V11.3, 303, 305.0
Smoking status	V15.82, 305.1
Drug abuse	304, 305.2-305.9
AIDS	042, V08

Table S2. Deyo's modification of Charlson's co-morbidity index (CCI).

**Condition**	**ICD-9-CM Codes**	**Charlson Score**
Myocardial infarction	410–410.9	1
Congestive heart failure	428 – 428.9	1
Peripheral vascular disease	433.9, 441–441.9, 785.4, V43.4	1
Cerebrovascular disease	430–438	1
Dementia	290–290.9	1
Chronic pulmonary disease	490–496, 500–505, 506.4	1
Rheumatologic disease	710.0, 710.1, 710.4, 714.0 – 714.2, 714.81, 725	1
Peptic ulcer disease	531–534.9	1
Mild liver disease	571.2, 571.5, 571.6, 571.4 –571.49	1
Diabetes	250–250.3, 250.7	1
Diabetes with chronic complications	250.4–250.6	2
Hemiplegia or paraplegia	344.1, 342–342.9	2
Renal disease	582–582.9, 583–583.7, 585, 586, 588–588.9	2
Any malignancy including leukemia and lymphoma	140–172.9, 174–195.8, 200–208.9	2
Moderate or severe liver disease	572.2–572.8	3
Metastatic solid tumor	196–199.1	6
AIDS	042–044.9	6
